# The Effect of Growth and Body Surface Area on Cardiopulmonary Exercise Testing: A Cohort Study in Preadolescent Female Swimmers

**DOI:** 10.3390/children10101608

**Published:** 2023-09-27

**Authors:** Vasileios T. Stavrou, Eleni Karetsi, Konstantinos I. Gourgoulianis

**Affiliations:** Laboratory of Cardio-Pulmonary Testing and Pulmonary Rehabilitation, Respiratory Medicine Department, Faculty of Medicine, University of Thessaly, 41100 Larissa, Greece; ekaretsi@uth.gr (E.K.); kgourg@uth.gr (K.I.G.)

**Keywords:** premenarcheal girls, biological maturation, oxygen pulse, oxygen breath

## Abstract

Background: The performance of young swimmers is the result of a multifactorial process that is influenced by anthropometric characteristics and biological maturation. The purpose of our study was to investigate the effect of stages of biological maturation and body surface area on cardiopulmonary fitness indicators in preadolescent female swimmers, for whom menstruation has not started. Methods: Thirty female preadolescent swimmers (age 13.4 ± 1.0 years) participated in this study. We recorded anthropometric and morphological characteristics, stages of biological maturation, and pulmonary function parameters, and the swimmers underwent cardiopulmonary exercise testing. Results: The cut-off was set for body surface area (BSA) at 1.6 m^2^ and for biological maturation stages at score 3. The BSA results showed differences in variabilities in maximal effort oxygen pulse (*p* < 0.001), oxygen uptake (*p* < 0.001), ventilation (*p* = 0.041), tidal volume (*p* < 0.001), and oxygen breath (*p* < 0.001). Tanner stage score results showed differences in variabilities in maximal effort breath frequency (*p* < 0.001), tidal volume (*p* = 0.013), and oxygen breath (*p* = 0.045). Biological maturation stages and BSA were correlated during maximal effort with oxygen breath (*p* < 0.001; *p* < 0.001), oxygen uptake (*p* = 0.002; *p* < 0.001), and oxygen pulse (*p* < 0.001; *p* < 0.001). Conclusions: In conclusion, the findings of our study showed that the girls who had a smaller body surface area and biological maturation stage presented lower values in maximal oxygen uptake and greater respiratory work.

## 1. Introduction

Cardiopulmonary exercise testing (CPET) is an examination that provides the assessment and response of the systems (the pulmonary, cardiovascular, hematopoietic, neuropsychological, and skeletal muscle systems), both at rest and especially during maximal exercise. CPET provides an assessment of comprehensive factors that are not adequately reflected by measuring the operation of the systems using individual instruments [1]. The key indicator of fitness to exercise through CPET is the maximum intake of O_2_, which reflects the respiratory, cardiovascular, muscular, and metabolic capacity of the body to absorb, transport, and consume oxygen, and is the component of multiple biological processes while expressing the upper limits of their adaptation during intense muscular effort; for this reason, this is an indicator of functional adaptability [2]. O_2_ uptake (V̇O_2_) is equal to the cardiac output [stroke volume (SV) x heart rate (HR)] times the difference between arterial (CaO_2_) minus the mixed venous O_2_ content (CvO_2max_), according to the equation by Fick [V̇O_2_ = (SV × HR) × (CaO_2_ − CvO_2_)], and is given in either absolute (mL·min^−1^) or relative values (mL·min^−1^·kg^−1^) [2].

The performance of young swimmers is the result of a multifactorial process that is influenced by anthropometric characteristics, training factors, and growth, and/or biological maturation [3]; biological maturation relates to sex, race, and ethnicity, as well as environmental factors and geographical area. Biological maturation is divided into three basic stages (delayed, synchronous, and early), is the transition from childhood to puberty, and is characterized by changes in body composition and physical performance [4]. Moreover, the athletes during training or competition are classified according to chronological age but with different biological maturation status, and that limits their performance due to reduced anaerobic enzymes, such as lactate dehydrogenase [5]. A study by Morais et al. [6] states that the fastest swimming speed is affected by the chest circumference, the hand surface area, and body surface area, which is associated with greater hydrodynamic resistance [7], which should be trained through the improvement of technique, mainly due to the period when the athletes have not completed their development. The maximum O_2_ intake in girls aged 12 to 16 years was observed to be constant in early maturing girls, but gradually increased from 12 to 17 years old in later maturing girls [8]. In females, the onset of puberty ranges from 8 to 13 years old, while the onset of menses is approximately 12.5 years old (within a range of 0.5 to 3 years), regardless of other parameters (e.g., race and ethnicity, etc.).

The purpose of our study was to investigate the effect of the stages of biological maturation and body surface area on cardiopulmonary fitness indicators in preadolescent female swimmers, for whom menstruation has not started. We hypothesized that the different stages of biological maturation and body surface area could affect the respiratory parameters during maximal cardiopulmonary exercise testing.

## 2. Materials and Methods

### 2.1. Participants

Thirty female preadolescent swimmers volunteered for this study (Table 1) from December 2018 to April 2023, without previous experience in cardiopulmonary exercise testing and pulmonary function tests. For all athletes, the inclusion criteria were ages ≥12 to ≤15 years; having >4 years of training, ≥60 min of training hours per week for the last two years, and training ≥4 times per week; competition experience in the National Swimming Championship ≥1 time; and without recent injury [9] and without myocardial hypertrophy [10]. The exclusion criteria were a lack of medical history and/or respiratory disorders [11], menstruation having not yet started [12], a sleep quality score with questionnaire Pittsburgh Sleep Quality Index > 5 [13], and previous SARS-CoV-2 infection [14]. This study’s protocol was approved by the Institutional Review Board (IRB)/Ethics Committee (EC) of the University of Thessaly, Greece (IRB/EC approval reference number: No. 58076/22.11.2018). All participants’ parents submitted a written consent form, in accordance with the Helsinki Declaration.

### 2.2. Data Collection Anthropometric and Morphological Characteristics

The study protocol initiated with the assessment of anthropometric and morphological characteristics. The body height was recorded using Seca 700 (Hamburg, Germany). Body mass, body composition, and total body water were assessed using whole-body bioelectrical impedance analysis (BIA) (Tanita MC-980, Arlington Heights, IL, USA) using a standard technique [15]. The body surface area (BSA) was calculated according to Mosteller’s [16] formula:

$$\mathrm{BSA}=\sqrt{\frac{\left(height\;\left(cm\right)\times body\;mass\;(kg)\right)}{3600}},$$

and body mass index (BMI) according to the formula:

$$\mathrm{BMI}=\frac{body\;mass\;(kg)}{height\;\left(m\right)\times height\;(m)}$$

The body composition measurements were conducted by the same operator in the morning, 09:30–10:30, and two hours after the wake up time of the participants according to what was recommended by the manufacturer and following all necessary accurate measurement guidelines [17]: (a) all athletes should not have exercised, or consumed energy drinks and nutrition supplements 24 h before; (b) at the day of examination, all participants should not have consumed any liquids or food at least 3 h before measurements; (c) the athletes were asked to empty their bowels and bladder at least 30 min before the measurement; and (d) all participants were in the standing position for at least 5 min before the measurement to redistribute the tissue fluids [17].

### 2.3. Biological Maturation

The stages of biological maturation were recorded according to the guidance of Emmanuel and Bokor [18].

Stage 1: No glandular breast tissue palpable;
Stage 2: Breast bud palpable under the areola;
Stage 3: Breast tissue palpable outside areola;
Stage 4: Areola elevated above the contour of the breast and;
Stage 5: Areolar mound recedes into single breast contour with areolar hyperpigmentation, papillae development, and nipple protrusion.

The assessment and recording of the maturation stage was performed by a female doctor and under the supervision of a parent of each child.

### 2.4. Pulmonary Function Test

All participants prior to CPET underwent standard spirometry and lung volume measurements using a MasterScreen-CPX pneumotachograph (VIASYS HealthCare, Hochberg, Germany) in line with ATS/ERS guidelines [19]. For each subject, three maximal flow-volume loops (the best trial was evaluated) were conducted, in the sitting position, to determine forced expiratory volume in the 1st s (FEV_1_), peak expiratory flow (PEF), and force vital capacity (FVC). Thoracic gas volume at expiratory reserve volume (ERV) and inspiratory capacity (IC) were measured. All trials were conducted with 40 s of rest between them and under the supervision of a pulmonologist.

### 2.5. Cardiopulmonary Exercise Testing

An electronic cycle ergometer (Ergoselect 100, Bitz, Germany) was used for all cardiopulmonary parameters (MasterScreen-CPX, VIASYS HealthCare, Hochberg, Germany), and a 12-lead ECG was also employed for heart rate (HR) monitoring (CareFusion, San Diego, CA, USA). The maximum heart rate was calculated according to the Tanaka [20] equation: HR_max_ = 207 − 0.7 × age (years). The CPET evaluation included 4 continuous stages: the 1st stage as a resting stage (duration: 2 min; speed: 0 rpm; and load: 0 watts), the 2nd stage as a warm-up and for familiarization with the procedures and equipment (duration: 3 min; speed: 55–60 rpm; and load: 0 watts), the 3rd stage as a main test with an increased work rate (duration: until exhausted; speed: 60–65 rpm; and load: started with 12 watts and increased by 15 watts/min with ramp pattern protocol); and the 4th stage as a recovery stage (duration: 5 min; speed: self-selected but under 50 rpm; and load: 0 watts). All predicted values and maximal loads were calculated according to Wasserman et al.’s [21] equation:

V̇O_2max_ [mL·min^−1^] = (height (cm) − age (yrs)) × 14

V̇O_2unloaded_ [mL·min^−1^] = 150 + (6 × body mass (kg))

$$\mathrm{Load}\;[\mathrm{work}\;\mathrm{rate}/\min^{-1}]=\frac{\left({V\;˙\;O}_{2\max}-{V\;˙\;O}_{2\mathrm{unloaded}}\right)}{100}$$

The oxygen pulse was calculated according to the Hansen [22] equation:

$$\frac{predicted\;{V\;˙\;O}_{2\max}}{predicted\;heart\;rate\;max}$$

All CPETs were performed by a clinical exercise physiologist and pulmonologist. All sessions were performed in the Laboratory of Cardiopulmonary Testing and Pulmonary Rehabilitation (University of Thessaly), with the environmental temperature at 25.2 ± 1.1 °C and a humidity of 35.2 ± 4.1%. The evaluation of the athletes was performed between 09:30 a.m. and 13:00 p.m. and during the specific preparatory training period (November to February).

### 2.6. Statistical Analysis

A power of 84% and confidence interval of 95% were adopted, with an estimated value for a type I error of 5% (G*Power software 3.1) for the sample size calculation in this study, and a value for 28 adolescent female swimmers was obtained. The data are presented as mean ± standard deviation (SD) and percentage (%). Data normality was assessed via the Kolmogorov–Smirnov one-sample test. Relationships between continuous variables were assessed using Pearson’s R correlation coefficients. The cut-off point for Tanner stages was set at a score of 3 [17] and that of body surface area was set as 1.6 m^2^ [23]. For all tests, a *p*-value of < 0.05 was considered statistically significant. The IBM SPSS 21 statistical package (SPSS Inc., Chicago, IL, USA) was used for all statistical analyses.

## 3. Results

**Figure 1:**
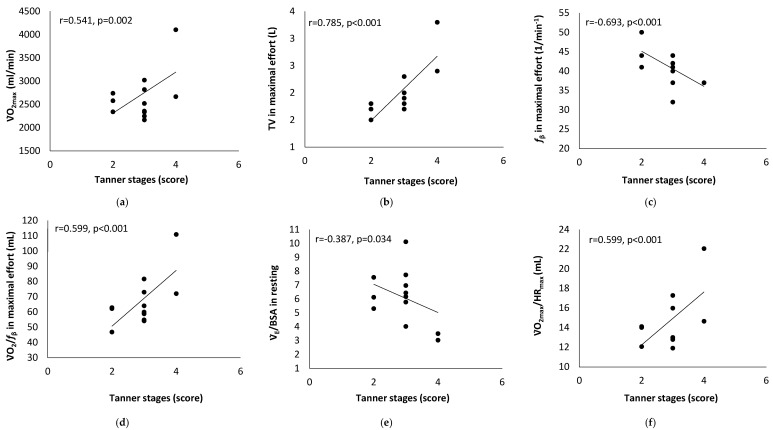
Correlation analysis results between Tanner stage score and maximal oxygen consumption (V̇O_2max_) (**a**), tidal volume (TV) (**b**), breath frequency (**c**), oxygen breath = ratio between oxygen consumption (V̇O_2_) and breath frequency (*f*_β_) (**d**), ratio between ventilation (V̇_E_) and body surface area (BSA) (**e**), and oxygen pulse = ratio between oxygen consumption (V̇O_2_) and heart rate (HR) (**f**).

**Figure 2:**
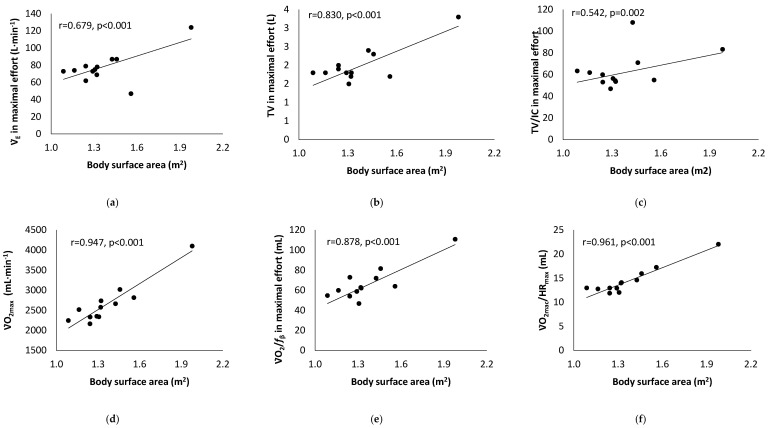
Correlation analysis results between body surface area and ventilation (V̇_E_) (**a**), tidal volume (TV) (**b**), ratio between tidal volume (TV) and inspiratory capacity (IC) (**c**), maximal oxygen consumption (V̇O_2_) (**d**), oxygen breath = ratio between oxygen consumption (V̇O_2_) and breath frequency (*f*_β_) (**e**), and oxygen pulse = ratio between oxygen consumption (V̇O_2_) and heart rate (HR) (**f**).

Table 1 presents the results of the athletes’ characteristics, and Table 2 presents the results of the cardiopulmonary exercise testing. A statistically significant correlation between Tanner stage score and cardiopulmonary fitness indicators is presented in Figure 1. Figure 2 presents the statistically significant correlation between body surface area and cardiopulmonary fitness indicators.

For the Tanner stage score, the cut-off point was set at 3 (*n* = 20: a score ≥ 3 versus *n* = 10: a score < 3). The Tanner stage score results showed differences in variabilities in maximal effort *f*_β_ (39.5 ± 2.9 versus 45.5 ± 5.0 1/min^−1^; *p* < 0.001), ΤV (2.1 ± 0.5 versus 1.7 ± 0.1 L; *p* = 0.013), and V̇O_2_/*f*_β_ (69.9 ± 19.6 versus 56.2 ± 8.1 mL; *p* = 0.045).

For body surface area, the cut-off point was set at 1.6 m^2^ (*n* = 5: ≥1.6 m^2^ versus *n* = 25: <1.6 m^2^). BSA results showed differences in variabilities in maximal effort V̇O_2_/HR (20.2 ± 2.2 versus 13.2 ± 1.2 mL; *p* < 0.001), V̇O_2_ (3589.6 ± 704.4 versus 2478.6 ± 241.8 mL·min^−1^; *p* < 0.001), V̇_E_ (93.2 ± 42.2 versus 75.5 ± 5.8 L·min^−1^; *p* = 0.041), V̇_E_/V̇CO_2_ (46.5 ± 10.0 versus 34.5 ± 3.0; *p* < 0.001), V̇_E_/V̇O_2_ (43.9 ± 14.7 versus 32.9 ± 3.1; *p* = 0.001), ΤV̇ (2.7 ± 0.9 versus 1.8 ± 0.2 L; *p* < 0.001), and V̇O_2_/*f*_β_ (92.2 ± 25.7 versus 60.0 ± 9.4 mL; *p* < 0.001).

## 4. Discussion

In this study, CPET variables were investigated in relation to the biological stages of maturation and body surface area in preadolescent swimmers who had not yet started their period. The main findings of our study are related to respiratory function and maximal oxygen uptake.

According to Papadimitriou et al., Greek girls showed an average menarche age of 12.27 years old [24]. Our study population was premenarcheal girls 13.4 years old. The menarcheal age is influenced by weight status and socioeconomic status; genetic and environmental factors also contribute, as well as the type, volume, and intensity of training at an early age [25], while competitive sport participation at ages 13–16 is associated with later menarche compared to no exercise at that age [26]. Previous studies have shown that swimming athletes who manage to stand out in a highly competitive environment are subjected to intense training sessions daily [27]. However, for many young swimmers who had not completed their development due to their age, the result was that there was a disproportionate relationship between burden and performance, including in competitive swimming, which was also influenced by parameters such as age, anthropometric and developmental characteristics, gender, and race [28]. According to Hayes et al., the maximal aerobic capacity in girls reaches a plateau from 14 years old onwards, while before 11 years old, endurance training does not affect aerobic capacity [29]. The athletes of our study had 2.8 ± 0.8 years of competition experience in the National Swimming Championship and a biological maturation stage score of 2.8 ± 0.7. Also, the participants had a maximal oxygen uptake of 45.1 ± 3.5 mL·kg^−1^·min^−1^ (126.2 ± 12.1% of predicted values), while the ventilatory anaerobic threshold was at 79.0 ± 10.0 percent of maximal effort, values that do not differ from previous studies (premenarcheal girls, 12 to 15 years old, V̇O_2max_ 40.4-to-65.0 mL·kg^−1^·min^−1^) [30,31,32,33,34], although we recommend that V̇O_2_ values are better expressed in terms of BSA (mL O_2_·m^2^·min^−1^) than in terms of weight (mL O_2_·kg^−1^·min^−1^) in children with low biological maturation. These values indicate a normal O_2_ transport and diffusion system at the cellular level and a normal rhythm of oxidative ATP production (i.e., cellular PO_2_ is greater than the critical value required to produce ATP at a maximal rate by oxidative phosphorylation in mitochondria) [35]. Still, the values for the end-tidal partial pressure of carbon dioxide and of oxygen, both at rest and at maximum effort, were within normal limits, i.e., the difference in the partial pressure of CO_2_ between arterial blood and end-expiratory air (P_ET_CO_2_) and the partial pressure of O_2_ in systemic arterial circulation (P_ET_O_2_) were interpreted as adequate [36].

Many times, swimming training is not separated into men and women but according to the swimming level of each athlete and chronological age [37]. Previous studies have adequately shown that women have smaller airway diameters, lung volumes, maximum expiratory flow, and diffusion surface areas compared to men [38]. The findings of our study showed that girls with a BSA <1.6 m^2^ have lower values in V̇O_2max_, V̇_Emax_, V̇O_2_/*f*_β_, and tidal volume compared to the largest female swimmers. According to Landgraff et al., [34] systematic high-volume training produces no additional effect on V̇O_2_max compared with a similar training volume which mainly aims at the development of motor skills, while the distribution of muscle fibers (fast- and slow-twitch) affects O_2_ uptake [39]. In addition, oxygen breath (V̇O_2_/*f*_β_) appeared to correlate with the cut-off for BSA and biological maturation stages. V̇O_2_/*f*_β_ is an index of respiratory adequacy and indicates the metabolic value of each breath. Moreover, the tidal volume indicator has been shown to be a useful parameter in children who are unable to perform to maximum exhaustion [40]. These relationships are probably associated with systematic changes in breathing patterns that act to optimize and increase endurance during maximal effort, while task failure occurred when these compensatory mechanisms were maximal [41,42].

The girls with a BSA < 1.6 m^2^ had lower values in CPET parameters (e.g., V̇O_2max_, V̇_Emax_, and V̇O_2_/*f*_β_) compared to the largest female swimmers, but those values were probably higher for their body surface area. This hypothesis probably interprets changes in core temperature and sweating during exercise, which are determined by metabolic heat production and body surface area [43]. We recently reported that breath frequency activates the parasympathetic system, particularly the vagus nerve innervating the lungs while swimmers have a breathing pattern, affected by swimming style [41,44]. As a result, the signals that are transferred through the vagus nerve when the athlete develops different breathing patterns due to smaller airway diameters, lung volumes, etc., and/or volitional hypoventilation (affected by swimming style), lead to increased PaCO_2_, decrease pH, and increase the frequency and the breadth of respiration in order to return respiration to normal through the procedure of hyperventilation due to need to provide O_2_ to tissues and excrete CO_2_. Although the respiratory values for V̇_E_/V̇O_2_ and V̇_E_/V̇CO_2_ were within normal limits, they showed a trend for an increased respiratory drive related to the amount and sensitivity of the central chemoreceptors and respiratory dead space and relate to an increase in brain PCO_2_, which stimulates breathing induced by metabolic acidosis [45]. In addition, the increase in V̇_E_/V̇O_2_ has been attributed to the maldistribution of pulmonary blood flow due to increased ventilation (Figure 2a) due to the effects of age and higher breathing efficiency (Figure 2b,c) [40].

Finally, previous studies have shown that low O_2_ uptake may be associated with a lower transport of blood O_2_ at the tissue level, due to low mitochondrial density and skeletal muscle inefficiency as reflected in people who have low muscle mass [9]. Finally, the O_2_ pulse (V̇O_2_/HR) showed a positive correlation with body surface area and biological maturation stages. The O_2_ pulse interprets the stroke volume and the difference of arterial and mixed venous blood [C(a-v)O_2_], while the values are influenced by age and body surface area [46]. Well-trained athletes can increase O_2_ pulse values by the duration of exercise due to increased skeletal muscle, i.e., mitochondrial oxidative capacity in capillaries leads to higher C(a-v)O_2_, according to Roca et al., [47] while Mazaheri et al. [48] recommend that the O_2_ pulse should be calculated relative to body surface area, because the amount of O_2_ pulse/BSA during submaximal exercise indicates central adaptations and, while at maximal exercise, indicates adaptations at the central and peripheral level [48].

### 4.1. Limitations and Strengths

Our results should be interpreted within our study’s limitations. Our study population was nested, and the reports of the athletes were from the region of Thessaly. The Thessaly region is in central Greece, which has 20 swimming clubs, and approximately 100 athletes every year participate in the age-group National Swimming Championship. Our study excluded, by design, girls whose menstruation had not started. The inclusion of such a group, however, would require a different study design that could address the perturbations introduced by the interrelationships between menstruation and breathing pattern, such as rising ventilation, dyspnea, and impaired ventilatory efficiency [49]. Another important limitation of our study is that recruitment involved adolescent female swimmers, and, therefore, may not apply to other athlete groups, e.g., volleyball, while the biological maturation assessment was from the same doctor. Another limitation is the lack of assessment of respiratory muscle strength, so we could not investigate respiratory fatigue [50].

### 4.2. Recommendations for Future Research

Our study presented interesting findings in cardiorespiratory fitness indices compared with body surface area and biological maturation in premenarcheal girls, despite the limitations we mention above. However, our recommendations for future research are: (a) long-term follow-up CPET and respiratory muscle strength testing in pre- and postmenarcheal female swimmers, (b) the relationship between CPET parameters and training volume, nutritional habits, sleep and chronotypes, and school performance in pre- and postmenarcheal female swimmers, and (c) a validity assessment of the oxygen uptake/body surface area (mL O_2_·m^2^·min^−1^) ratio in children with low biological maturation.

## 5. Conclusions

In conclusion, the findings of our study showed that the girls who underwent CPET and had a smaller body surface area and low biological maturation stage presented lower values in V̇O_2max_ and greater respiratory work. CPET has practical applications for the evaluation and guidance of the athlete according to their available capabilities at any given time. Coaches should encourage athletes to be evaluated with CPET so that the information provided may help to structure training in a targeted manner, especially for athletes who have not completed their development.

## Figures and Tables

**Table 1:** Athletes’ characteristics. Data are expressed as mean ± standard deviation and number (*n*).

		Mean ± Sd
Age	years	13.4 ± 1.0
Body mass index	kg/m^2^	21.0 ± 2.5
Body surface area	m^2^	1.4 ± 0.2
Lean body mass	kg	49.4 ± 4.6
Total body water	%	51.9 ± 3.0
Swimming style	FR	100 m (*n* = 8), 200 m (*n* = 5)
	BK	100 m (*n* = 6), 200 m (*n* = 2)
	BR	100 m (*n* = 4), 200 m (*n* = 2)
	BF	200 m (*n* = 1),
	IM	200 m (*n* = 2)
Tanner scale	score	2.8 ± 0.7
PSQI	score	1.3 ± 2.1
FEV_1_	L (% of pred)	3.8 ± 0.8 (125.8 ± 11.9)
FVC	L (% of pred)	4.6 ± 0.7 (122.7 ± 10.6)
PEF	L (% of pred)	6.7 ± 1.2 (108.1 ± 4.1)
ERV	L (% of pred)	1.9 ± 0.8 (148.1 ± 54.0)
IC	L (% of pred)	3.2 ± 0.5 (123.5 ± 17.8)

Abbreviations: BF = butterfly; BK = backstroke; BR = breaststroke; ERV = expiratory reserve volume; FEV_1_ = forced expiratory volume in 1st s; FR = freestyle; FVC = forced vital capacity; IC = inspiratory capacity; IM = individual medley; PEF = peak expiratory force; PSQI = Pittsburgh Sleep Quality Index.

**Table 2:** Cardiopulmonary exercise testing results. Data are expressed as mean ± standard deviation and percentage.

		Resting	Maximal Effort
V̇O_2_	mL·min^−1^	244.5 ± 69.9	2663.8 ± 542.4
	mL·kg^−1^·min^−1^	4.2 ± 1.3	45.1 ± 3.5
	% of pred		126.2 ± 12.1
V̇CO_2_	mL·min^−1^	189.7 ± 39.9	2834.2 ± 735.0
V̇_E_/MVV	%	6.4 ± 1.8	59.3 ± 10.0
IC/TV	%	15.9 ± 5.9	61.5 ± 13.2
V̇_E_/V̇O_2_		29.9 ± 6.5	34.7 ± 7.4
V̇_E_/V̇CO_2_		23.1 ± 2.3	36.5 ± 6.5
*f*_β_	1·min^−1^	17.9 ± 3.6	41.5 ± 4.3
P_ET_O_2_	mmHg	111.7 ± 7.5	111.8 ± 3.3
P_ET_CO_2_	mmHg	30.0 ± 4.0	38.3 ± 3.3
Heart rate	bpm (% of pred)	84.3 ± 7.4 (41.0 ± 3.7)	185.4 ± 9.1 (90.2 ± 4.4)
Load	watts·kg^−1^		3.4 ± 0.5
CPET duration (3rd stage)	min		12.4 ± 0.9

Abbreviations: *f*_β_ = breath frequency; IC/TV = ratio between inspiratory capacity (IC) and tidal volume (TV); P_ET_CO_2_ = end-tidal partial pressure of carbon dioxide; P_ET_O_2_ = end-tidal partial pressure of oxygen; V̇CO_2_ = carbon dioxide production; V̇_E_/MVV = ratio between maximal ventilation during exercise (VE) and maximum voluntary ventilation (MVV); V̇_E_/V̇CO_2_ = ventilatory equivalent for carbon dioxide; V̇_E_/V̇O_2_ = ventilatory equivalent for oxygen; and V̇O_2_ = oxygen consumption.

## Data Availability

All data are available upon request.
